# Photocontrolled Exposure of Pro‐apoptotic Peptide Sequences in LOV Proteins Modulates Bcl‐2 Family Interactions

**DOI:** 10.1002/cbic.201500469

**Published:** 2015-11-20

**Authors:** Robert J. Mart, Dilruba Meah, Rudolf. K. Allemann

**Affiliations:** ^1^ School of ChemistryCardiff UniversityMain BuildingPark PlaceCardiffCF10 3ATUK

**Keywords:** apoptosis, photo-uncaging, protein engineering, protein–protein interactions

## Abstract

LOV domains act as biomolecular sensors for light, oxygen or the environment's redox potential. Conformational changes upon the formation of a covalent cysteinyl flavin adduct are propagated through hydrogen‐bonding networks in the core of designed hybrid phototropin LOV2 domains that incorporate the Bcl homology region 3 (BH3) of the key pro‐apoptotic protein BH3‐interacting‐domain death agonist (BID). The resulting change in conformation of a flanking amphiphilic α‐helix creates a light‐dependent optogenetic tool for the modulation of interactions with the anti‐apoptotic B‐cell leukaemia‐2 (Bcl‐2) family member Bcl‐x_L_.

Light‐oxygen‐voltage (LOV) domains are molecular switches that act as internal sensors of oxygen, redox potential and light in cells.^1^, ^2^, ^3^ Some LOV domains function as reversible photoswitches^4^ and underpin a range of blue‐light responses in plants, fungi and bacteria including phototropism^5^, ^6^ and regulation of the circadian rhythm.^7^ LOV photosensors share a common mechanism by which a noncovalently bound flavin cofactor absorbs blue light (450–475 nm) to enter an excited electronic state; this leads to the formation of a covalent adduct between flavin mononucleotide (FMN) and the sulfur atom of a cysteine residue,^8^ and significant conformational changes occur.^9^ The FMN adduct spontaneously reverts to its noncovalently bonded dark state with rates that reflect the function of the individual LOV domain. Phototropin was one of the first blue‐light receptors discovered in plants, and LOV2 from *Avena sativa* (*As*LOV2) has previously been used for photoprotein engineering. In its dark form, the C‐terminal Jα helix of *As*LOV2 is tightly bound to the β‐sheet (Figure 1), but upon light‐activated adduct formation, the 20 residues of the C‐terminal Jα helix are displaced from the β‐sheet, thereby exposing the amphiphilic helix (Figure 2).^10^, ^11^, ^12^

**Figure 1:**
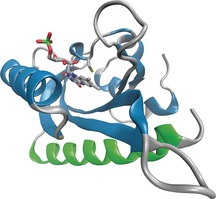
Overall structure of *Avena sativa* Phototropin 1 LOV2 domain (PDB ID: 2V1A)^12^ with the Jα helix in green.

**Figure 2:**
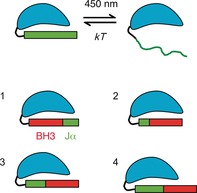
Cartoon illustrating the dark and irradiated forms of *As*LOV2 and the four hybrids between *As*LOV2 and BID, LOV‐BID1 to LOV‐BID4, with different locations of the BH3 recognition element of BID (red) within the Jα helix of *As*LOV2 (green).

LOV domains have been used to create light‐responsive DNA‐binding motifs^13^, ^14^, ^15^ and transcriptional activators,^16^, ^17^ as well as to control the activity of enzymes^18^, ^19^ and the subcellular location^20^ and degradation rates^21^ of proteins by domain fusion or insertion. We have previously modified peptide sequences from proapoptotic proteins with azobenzene crosslinkers to create biomolecular nanoswitches (BNs), whose conformations and binding properties change in response to light.^22^, ^23^, ^24^ A LOV‐derived protein of equivalent functionality could be genetically encoded and photoactivated in vivo through transient expression or gene integration. A LOV/caspase 7 hybrid has previously been shown to cause cell death; however, overexpression of Bcl‐2, which is common in many types of cancer cells, diminished its proapoptotic effect.^25^

Previous work has sought to maximise the dynamic range between the light‐ and dark‐state affinities of LOV–peptide fusions,^21^, ^26^ but controlling potentially irreversible apoptotic processes with an expressed protein requires stringent “caging” of the active epitope. Caging efficiency is affected by the position of the binding epitope in the Jα helix; residues incorporated closer to the body of the protein are better caged in the dark state but pay a steric penalty in the light state. Well‐characterised protein–protein interaction motifs have been introduced into LOV domains to generate generic photoassociation tools. Incorporating an amino acid sequence that is strongly bound by PSD‐95/discs large/zona occludens 1 (PDZ) domains into sites between residues 540 and 545 of the Jα helix of *As*LOV2 (Table S1) led to proteins with increased affinities for PDZ in the light‐activated state.^27^ Introducing the recognition sequence at residue 542 led to the widest dynamic range between dark‐ and light‐state affinities. A protein database search revealed that peptide sequences similar to *As*LOV2 Jα have been crystallised bound to interacting partners.^28^ Elements of one such sequence, the SsrA peptide, were incorporated at residues 523, 535, 538 and 542 of the Jα helix of *As*LOV2. The abilities of these proteins to bind SspB, the cognate partner of SsrA, were compared by fluorescence polarisation; whilst the sequence inserted at 538 showed the tightest binding affinity, insertion at 542 led to the greatest difference between light and dark states. Proteins could be marked for light‐dependent proteasomal degradation by inserting a four‐amino acid degrons, RRRG, at residue 543 of *As*LOV2.^21^ A sequence from a cAMP‐dependent kinase inhibitor was inserted into the loop preceding the *As*LOV2 Jα, but this change led to inhibition of the target enzyme even in the dark state.^29^ In contrast, appending the inhibitor sequence to the *As*LOV2 Jα at residue 452 led to light‐dependent inhibition of the target kinase. Taken together, these results suggest that only a rather narrow region of the Jα region of *As*LOV2 can be used to generate effective photocaged protein hybrids. This is emphasised by fusions of LOV to Rac1, a GTPase, in which the addition or removal of single amino acids drastically alters dark‐state caging of the GTPase^30^

The key structural recognition element for proapoptotic proteins is the Bcl homology 3 region (BH3), which binds as an α‐helix in a shallow groove found on the surface of Bcl family proteins. BH3 recognition elements are much longer (21 residues rather than 7–9 residues, Table S1) than the sequences that have previously been incorporated into the Jα helix of *As*LOV2, and hydrophobic side chains from four separate turns contribute strongly to the binding affinity. In addition, several intervening residues make highly conserved interactions or dictate specificity for anti‐apoptotic protein subfamilies.^31^ We chose the BH3 domain of BID, a broadly acting pro‐apoptotic protein, as a model sequence for changes to the Jα of *As*LOV2. Key residues of the Jα helix in *As*LOV2 determine its dark‐state structure (Figure 1); G528A and N538E mutants show increased helix docking in the dark state, whereas *As*LOV2‐I532E and *As*LOV2‐A536E create “pseudo‐light” mutants by compromising Jα docking.^10^, ^27^ To design an efficient *As*LOV2‐based optogenetic tool that targets the heterodimers of the Bcl‐2 family, the hydrophobic character of residues I532, A536 and I539 of *As*LOV needed to be preserved, and D540, a residue that makes an important electrostatic interaction to the core LOV domain,^10^ needed to be retained (Table 1).

**Table 1:** Amino acid sequences of the Jα‐helix sequences (BID BH3‐type sequences underlined, altered residues in italics). The LOV‐BID peptide consists of the bold residues in LOV‐BID1 with an additional A523C change to accommodate a fluorophore.

Protein^[a]^	Partial sequence [Jα region]	*T*_m_	*t*_1/2_^UV^
		[°C]	[min]
His_6_‐*As*LOV2‐V416I	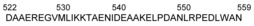		11.4±0.12
BID BH3	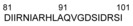		
LOV‐BID1	**D**A**AE** ***DI*** **GV** ***NIARHL*** **A** ***QVG*** **D** ***SIDRSI***PDANLRPEDLWAN	66	10.4±0.05
LOV‐BID2	DAAEREGVMLIK*DI* A *RN* ID *R* A *LA* E *VGDSIDRSI*	55	8.60±0.05
LOV‐BID3	DAAEREGVMLIKKTA*DI* ID *N* AA *R* EL *AQVGDSIDRSI*	51	7.50±0.17
LOV‐BID4	DAAEREGVMLIKKTAENID *I* A *RNIARHLAQVGDSIDRSI*	49	7.80±0.75

[a] All LOV‐BID proteins described include the V416I mutation to stabilise the cysteinyl‐FMN adduct.

Despite these compromises, a fluorescently labelled peptide corresponding to the proposed Jα region of the hybrid LOV–BID1 (Table 1) showed only a twofold reduction in affinity for Bcl‐x_L_ (*K*_D_=46±2.6 nm) relative to the parent sequence (Table S4). A plasmid containing DNA encoding *As*LOV2 fused to the C terminus of a domain used for affinity purification, hisactophilin‐C49S,^32^ was used to construct hybrids between *As*LOV2 and BID BH3. Although wild‐type Hisact‐*As*LOV2 relaxed with a half‐life of approximately 1 min after photoactivation, a mutant with a different hydrophobic side chain near the active site, Hisact‐*As*LOV2‐V416I, had a significantly longer‐lived (*t*_1/2_=7.7 min) photoactivated state (Table S5).^33^, ^34^ The hisactophilin prosthetic domain was replaced by a His‐tag, and BH3‐like sequences were added at different positions of the Jα helix (Table 1) to generate LOV‐BID1–4. Solutions of these hybrids were exposed to 455 nm light generated by an LED, and the recovery of FMN absorbance at 455 nm was followed by UV spectroscopy; the ellipticity change at 222 nm in the circular dichroism spectrum was measured by detecting the recovery of α‐helicity after exposure to blue light. All four proteins were photoresponsive, and the half‐lives obtained from CD and UV measurements were broadly similar, varying between 10.4 (LOV‐BID1) and 7.5 min (LOV‐BID3; Table 1 and S5).

The thermal stabilities of the hybrids, as measured by CD spectroscopy, decreased from LOV‐BID1 to LOV‐BID4 (Table 1). The degree of structural change shown by CD spectroscopy upon photoactivation is strongly reduced in LOV‐BID1 compared to *As*LOV2‐V416I (Figure 3). Comparison of the mean residue ellipticities of photoactivated states of *As*LOV2 (V416I) and LOV‐BID1 at 222 nm suggests a decreased helicity in the dark state, as both the Jα helix in the light state of wild‐type *As*LOV2^34^ and BID BH3 peptides^22^ are unstructured. LOV‐BID2 shows little difference in structure between the dark and photoactivated states, but single‐wavelength monitoring could be used to fit a decay curve to a change at 222 nm. As the value obtained matches the half‐life of the other hybrids, it appears the Jα helix is either disordered in the dark state or maintains helicity in the photoactivated state rather than cysteinyl‐FMN adduct formation becoming decoupled from structural changes. Much larger changes were observed in the CD spectra for light‐activated and dark‐state LOV‐BID1, LOV‐BID3 and LOV‐BID4 samples.

**Figure 3:**
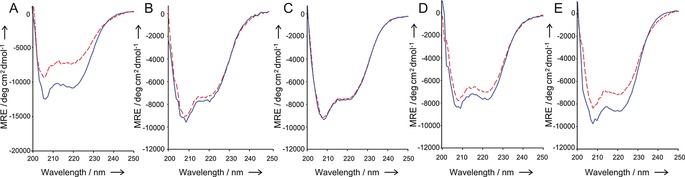
Circular dichroism spectra of proteins in the dark‐adapted (blue) and lit states (red) after 30 s of irradiation with a 1 W 455 nm LED in sodium phosphate (50 mm, pH 7.5) buffer containing sodium chloride (10 mm). A) *As*LOV2 (V416I) B) LOV‐BID1 C) LOV‐BID2 D) LOV‐BID3 E) LOV‐BID4.

A fluorescence anisotropy assay was used to measure the ability of the hybrids LOV‐BID1–4 in their light‐activated and dark states to target fluorescently labelled loop‐truncated Bcl‐x_L_.^22^, ^23^ As expected, no binding was observed for *As*LOV2 (V416I) either before or after photoactivation (Figure 4). LOV‐BID hybrids on the other hand, showed high‐nanomolar affinities for Bcl‐x_L_ in their photoactivated states. Light‐state dissociation constants decreased as the length of hybrid Jα helix increased (Table 2); this possibly reflects the increased accessibility of the BH3 motif. No binding to Bcl‐x_L_ could be measured in the dark‐adapted states of LOV‐BID1, LOV‐BID3 and LOV‐BID4, but LOV‐BID2 bound to Bcl‐x_L_ in the dark with threefold reduced affinity compared to that in its photoactivated state. This suggests that the Jα region of LOV‐BID2 is poorly caged in the dark state rather than remaining structured in the light‐activated state.

**Figure 4:**
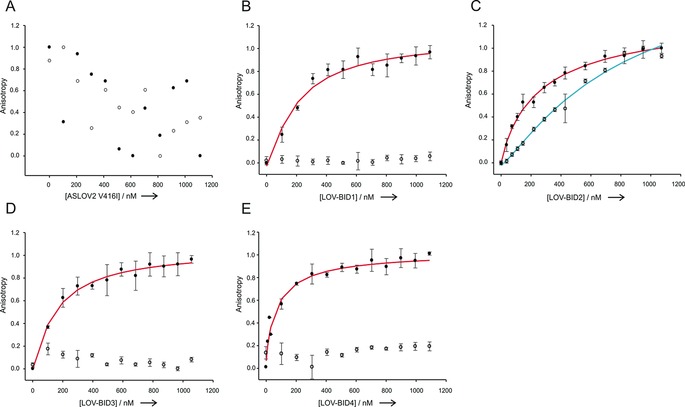
Normalised fluorescent anisotropy binding curves of proteins to TAMRA‐labelled Bcl‐x_L_ (S2C) (10 nm) in sodium phosphate buffer (50 mm, pH 7.5) containing NaCl (10 mm) at 15 °C to minimise relaxation during the experiment in the dark‐adapted (○) and lit states (•) after 30 s irradiation of with a 1 W, 455 nm LED A) *As*LOV2 (V416I); B) LOV‐BID1; C) LOV‐BID2; D) LOV‐BID3 and E) LOV‐BID4.

**Table 2:** Binding affinities of LOV proteins in their dark state and lit states.

Protein	Dark‐adapted *K*_D_ [nm]	Light‐activated state *K*_D_ [nm]
*As*LOV2 (V416I)	n.d.	n.d.
LOV‐BID1	n.d.	216±16
LOV‐BID2	998±111	271±14
LOV‐BID3	n.d.	167±3
LOV‐BID4	n.d.	89±5

Without calculating the dark‐state affinities, it is impossible to calculate the dynamic range of LOV‐BID1, LOV‐BID3 and LOV‐BID4. However, the best dynamic range obtained in previous peptide experiments was a 23‐fold difference in affinities for an *i,i*+4 azobenzene‐conjugated BID peptide.^22^ An equivalent dynamic range would equate to a dark state affinity of approximately 2 μm for LOV‐BID4, which it greatly exceeds. Even without further mutations, such as those used elsewhere to modify the strength of Jα‐helix docking, the switching magnitude of the LOV‐BID proteins reported here is better than in many previous reports^21^, ^27^, ^28^, ^29^ and similar to the best reported values for LOV‐SsrA variants optimized by phage display (36‐ and 58‐fold).^35^ The penalty for embedding the BH3 sequence closer to the core of the LOV domain is relatively low (∼2.5‐fold) compared to LOV‐SsrA proteins (∼16‐fold).^28^ This might reflect the structure of the binding site of the target protein; Bcl‐x_L_ presents a shallow groove across one face with space at either end for overhanging protein.

Incorporating Bid BH3‐derived sequences into the Jα helix of *As*LOV2 did not alter the photochemistry of the LOV domain; it was generally well tolerated, resulting in proteins that underwent conformational changes in response to irradiation with blue light. The affinity of the embedded BH3 sequences for Bcl‐x_L_ was dependent on the conformational state of the LOV‐BID fusions, which offer significant potential as optically controlled intracellular modulators of protein–protein interactions. The relative ease of integration of peptide sequences based on amphiphilic helices (in contrast with previous work incorporating more polar sequences) suggests wider applications of LOV photoswitches to rapidly and reversibly control protein levels and activities with light at the post‐translational level. Photo‐exposure of peptide epitopes in LOV domain hybrids introduced into transiently or stably transfected cells will generate potent optogenetic tools that avoid the difficulties of trafficking peptides across the cell membranes and offer a complementary approach to the use of azobenzene photoswitches.

## Supporting information

As a service to our authors and readers, this journal provides supporting information supplied by the authors. Such materials are peer reviewed and may be re‐organized for online delivery, but are not copy‐edited or typeset. Technical support issues arising from supporting information (other than missing files) should be addressed to the authors.

Supplementary

## References

[1] C. L. Partch , K. H. Gardner , J. Cell. Physiol. 2010, 223, 553–557. 20112293 10.1002/jcp.22067 PMC2872778

[2] S. Crosson , K. Moffat , Proc. Natl. Acad. Sci. USA 2001, 98, 2995–3000. 11248020 10.1073/pnas.051520298 PMC30595

[3] K. S. Conrad , C. C. Manahan , B. R. Crane , Nat. Chem. Biol. 2014, 10, 801–809. 25229449 10.1038/nchembio.1633 PMC4258882

[4] T. E. Swartz , S. B. Corchnoy , J. M. Christie , J. W. Lewis , I. Szundi , W. R. Briggs , R. A. Bogomolni , J. Biol. Chem. 2001, 276, 36493–36500. 11443119 10.1074/jbc.M103114200

[5] W. R. Briggs , J. M. Christie , Trends Plant Sci. 2002, 7, 204–210. 11992825 10.1016/s1360-1385(02)02245-8

[6] W. R. Briggs , T. S. Tseng , H. Y. Cho , T. E. Swartz , S. Sullivan , R. A. Bogomolni , E. Kaiserli , J. M. Christie , J. Integr. Plant Biol. 2007, 49, 4–10.

[7] N. Huang , Y. Chelliah , Y. Shan , C. A. Taylor , S. H. Yoo , C. Partch , C. B. Green , H. Zhang , J. S. Takahashi , Science 2012, 337, 189–194. 22653727 10.1126/science.1222804 PMC3694778

[8] C. W. M. Kay , E. Schleicher , A. Kuppig , H. Hofner , W. Rudiger , M. Schleicher , M. Fischer , A. Bacher , S. Weber , G. Richter , J. Biol. Chem. 2003, 278, 10973–10982. 12525505 10.1074/jbc.M205509200

[9] J. P. Zayner , C. Antoniou , T. R. Sosnick , J. Mol. Biol. 2012, 419, 61–74. 22406525 10.1016/j.jmb.2012.02.037 PMC3338903

[10] S. M. Harper , J. M. Christie , K. H. Gardner , Biochemistry 2004, 43, 16184–16192. 15610012 10.1021/bi048092i

[11] S. M. Harper , L. C. Neil , K. H. Gardner , Science 2003, 301, 1541–1544. 12970567 10.1126/science.1086810

[12] A. S. Halavaty , K. Moffat , Biochemistry 2007, 46, 14001–14009. 18001137 10.1021/bi701543e

[13] D. Strickland , K. Moffat , T. R. Sosnick , Proc. Natl. Acad. Sci. USA 2008, 105, 10709–10714. 18667691 10.1073/pnas.0709610105 PMC2504796

[14] A. I. Nash , R. McNulty , M. E. Shillito , T. E. Swartz , R. A. Bogomolni , H. Luecke , K. H. Gardner , Proc. Natl. Acad. Sci. USA 2011, 108, 9449–9454. 21606338 10.1073/pnas.1100262108 PMC3111320

[15] G. Rivera-Cancel , L. B. Motta-Mena , K. H. Gardner , Biochemistry 2012, 51, 10024–10034. 23205774 10.1021/bi301306t PMC3531242

[16] L. R. Polstein , C. A. Gersbach , J. Am. Chem. Soc. 2012, 134, 16480–16483. 22963237 10.1021/ja3065667 PMC3468123

[17] X. Wang , X. J. Chen , Y. Yang , Nat. Methods 2012, 9, 266–269. 22327833 10.1038/nmeth.1892

[18] J. Lee , M. Natarajan , V. C. Nashine , M. Socolich , T. Vo , W. P. Russ , S. J. Benkovic , R. Ranganathan , Science 2008, 322, 438–442. 18927392 10.1126/science.1159052 PMC3071530

[19] B. Schierling , A. Pingoud , Bioconjugate Chem. 2012, 23, 1105–1109. 10.1021/bc3001326 22559722

[20] D. Niopek , D. Benzinger , J. Roensch , T. Draebing , P. Wehler , R. Eils , B. Di Ventura , Nat. Commun. 2014, 5, 4404. 25019686 10.1038/ncomms5404 PMC4104460

[21] K. M. Bonger , R. Rakhit , A. Y. Payumo , J. K. Chen , T. J. Wandless , ACS Chem. Biol. 2014, 9, 111–115. 24180414 10.1021/cb400755b PMC3906921

[22] S. Kneissl , E. J. Loveridge , C. Williams , M. P. Crump , R. K. Allemann , ChemBioChem 2008, 9, 3046–3054. 19012295 10.1002/cbic.200800502

[23] P. Wysoczanski , R. J. Mart , E. J. Loveridge , C. Williams , S. B.-M. Whittaker , M. P. Crump , R. K. Allemann , J. Am. Chem. Soc. 2012, 134, 7644–7647. 22515821 10.1021/ja302390a

[24] R. J. Mart , R. J. Errington , C. L. Watkins , S. C. Chappell , M. Wiltshire , A. T. Jones , P. J. Smith , R. K. Allemann , Mol. Biosyst. 2013, 9, 2597–2603. 23942570 10.1039/c3mb70246d

[25] E. Mills , X. Chen , E. Pham , S. Wong , K. Truong , ACS Synth. Biol. 2012, 1, 75–82. 23651071 10.1021/sb200008j

[26] D. Strickland , X. Yao , G. Gawlak , M. K. Rosen , K. H. Gardner , T. R. Sosnick , Nat. Methods 2010, 7, 623–626. 20562867 10.1038/nmeth.1473 PMC2914111

[27] D. Strickland , Y. Lin , E. Wagner , C. M. Hope , J. Zayner , C. Antoniou , T. R. Sosnick , E. L. Weiss , M. Glotzer , Nat. Methods 2012, 9, 379–384. 22388287 10.1038/nmeth.1904 PMC3444151

[28] O. I. Lungu , R. A. Hallett , E. J. Choi , M. J. Aiken , K. M. Hahn , B. Kuhlman , Chem. Biol. 2012, 19, 926–926. 10.1016/j.chembiol.2012.02.006 PMC3334866 22520757

[29] J. J. Yi , H. Wang , M. Vilela , G. Danuser , K. M. Hahn , ACS Synth. Biol. 2014, 3, 788–795. 24905630 10.1021/sb5001356 PMC4277778

[30] Y. I. Wu , D. Frey , O. I. Lungu , A. Jaehrig , I. Schlichting , B. Kuhlman , K. M. Hahn , Nature 2009, 461, 104–108. 19693014 10.1038/nature08241 PMC2766670

[31] L. Chen , S. N. Willis , A. Wei , B. J. Smith , J. I. Fletcher , M. G. Hinds , P. M. Colman , C. L. Day , J. M. Adams , D. C. Huang , Mol. Cell 2005, 17, 393–403. 15694340 10.1016/j.molcel.2004.12.030

[32] W. Eisenreich , M. Joshi , B. Illarionov , G. Richter , W. Römisch-Margl , F. Müller , A. Bacher , M. Fischer , FEBS J. 2007, 274, 5876–5890. 17944933 10.1111/j.1742-4658.2007.06111.x

[33] J. M. Christie , S. B. Corchnoy , T. E. Swartz , M. Hokenson , I. S. Han , W. R. Briggs , R. A. Bogomolni , Biochemistry 2007, 46, 9310–9319. 17658895 10.1021/bi700852w

[34] B. D. Zoltowski , B. Vaccaro , B. R. Crane , Nat. Chem. Biol. 2009, 5, 827–834. 19718042 10.1038/nchembio.210 PMC2865183

[35] G. Guntas , R. A. Hallett , S. P. Zimmerman , T. Williams , H. Yumerefendi , J. E. Bear , B. Kuhlman , Proc. Natl. Acad. Sci. USA 2015, 112, 112–117. 25535392 10.1073/pnas.1417910112 PMC4291625

