# Supplementary material for: Photocontrolled Exposure of Pro‐apoptotic Peptide Sequences in LOV Proteins Modulates Bcl‐2 Family Interactions
Source: Chembiochem. 2015 Nov 20;17(8):698–701. doi: 10.1002/cbic.201500469 (PMC5063126; doi:10.1002/cbic.201500469)
Supplement: Supplementary file 1 — Supplementary [file CBIC-17-698-s001.pdf]

CHEMBIOCHEM

Supporting Information

Photocontrolled Exposure of Pro-apoptotic Peptide Sequences in LOV Proteins Modulates Bcl-2 Family Interactions

Robert J. Mart, Dilruba Meah, and Rudolf. K. Allemann*[a]

cbic_201500469_sm_miscellaneous_information.pdf

1 Protein Design

Table S1: *The position of protein-protein interaction epitopes in the C-terminal helices of Avena sativa Phototropin1 LOV2 domain in previous studies compared to LOV-BID1. Residues highlighted in grey form a hydrophobic stripe docking the Jα helix to the β-sheet in the dark state. Bold residues indicate alterations from the wild type sequence and binding epitopes are underlined.*

Protein	*Sequence*
	522 530 540 550 559
*AS*LOV2	DAAEREGVMLIKKTAENIDEAAKELPDANLRPEDLWAN
TULIP5[a]	DAAEREGVMLIKKTAENI**SSADTWV**
TULIP4[a]	DAAEREGVMLIKKTAENID**SSADTWV**
TULIP3[a]	DAAEREGVMLIKKTAENIDE**SSADTWV**
TULIP2[a]	DAAEREGVMLIKKTAENIDEA**SSADTWV**
TULIP1[a]	DAAEREGVMLIKKTAENIDEAA**SSADTWV**
LOV-SsrAN[b]	DAA**ND**E**AY**MLIKKTAE**E**IDEAAKEL
LOV-SsrAM[b]	DAAERE**A**VMLIKK**AAND**I**NY**AAKEL
LOV-SsrAC[b]	DAAERE**A**VMLIKKTAE**E**IDEAA**NDENY**F
oLID[c]	DAAERE**A**VMLIKKTAE**E**IDEAA**NDENY**F
iLID[c]	**G**AAERE**A**V**C**LIKKTA**FQ**IAEAA**NDENY**F
PA-PKI (14-22)[d]	DAAEREGVMLIKKTAENIDE**GRTGRRNAI**
LOV24 (Degron)[e]	DAAEREGVML**A**KKTAENIDEAA**RRRG**

[a] Strickland et al.[1] *[b] Lungu et al.*[2] *[c]* Guntas *et al.*[3] These proteins also include additional changes to the loop preceeding the Jα *[d]* Yi *et al.*[4] *[e]* Bonger *et al.*[5]

Table S2: *Conserved residues amongst proapoptotic BH3 proteins*

Parent protein	*BH3 Sequence*
BID	DIIRN**I**ARH**L**AQ**VGD**S**I**DRSI
BAK	STMGQ**V**GRQ**L**AI**IGD**D**I**NRRY
BAD	WAAQR**Y**GRE**L**RR**MSD**E**F**VDSF
BIM	RPEIW**I**AQE**L**RR**IGD**E**F**NAYY
BAX	ASTKK**L**SEC**L**KR**IGD**E**L**DSNM

2 LOV-BID and BID BH3 Peptides

All peptides were synthesized according to standard fluorenylmethylcarbamoyl (Fmoc) solid phase synthesis protocols using a CEM Liberty microwave-assisted peptide synthesizer. The amino acids were protected with trityl (Trt), tert-butyl (tBu), butoxycarbonyl (Boc) or 2,2,4,6,7-pentamethyldihydrobenzofurane (Pbf) sidechain protecting groups as required, O-benzotriazole-N,N,N',N'-tetramethyluroniumhexafluorophosphate (HBTU), hydroxyl-benzotriazole (HOBt), N-methylpyrrolidinone (NMP) and dimethylformamide (DMF) were purchased from AGTC Bioproducts. Dichloromethane (DCM), trifluoroacetic acid (TFA) and diethyl ether were sourced from Fisher. Piperidine, acetic anhydride, triisopropylsilane (TIS), N,N-diisopropylethylamine (DIEA), 4-(2-hydroxyethyl)-1-piperizineethanesulfonic acid (HEPES) and triscarboxyethylphosphine (TCEP) were purchased from Sigma Aldrich. Rink Amide resin (0.72 mmol/g) and 5(6)-carboxyfluorescein were purchased from NovaBioChem.

Peptide samples were purified by reverse phase HPLC (Dionex) using a Phemonenex Gemini C18 column (10 µm, 110 A, 10×250 mm) with an water/gradient from 0 % to 100 % acetonitrile (0.1 % TFA) containing in water (0.1 % TFA) over 50 min at a flow rate of 5 mL/min (Figures 10 and 15), peaks were collected, identified by MALDI-TOF MS (Matrix: α-cyano-hydroxy-cinnamic acid in 1:1 ratio of CH_3CN/H_2O) and the samples were freeze-dried and kept at -20 °C until further use.

The purity of peptides was confirmed using analytical HPLC using an Acclaim Dionex Ultimate-3000 equipped with a Acclaim C18 column (3 µm, 120 A, 4.6×150 mm) with a gradient from 100% water (0.1% TFA) to 100% acetonitrile (0.1% TFA) over 50 minutes at a flow rate of 1 mL/minute. Peptides were identified using MALDI-TOF MS.

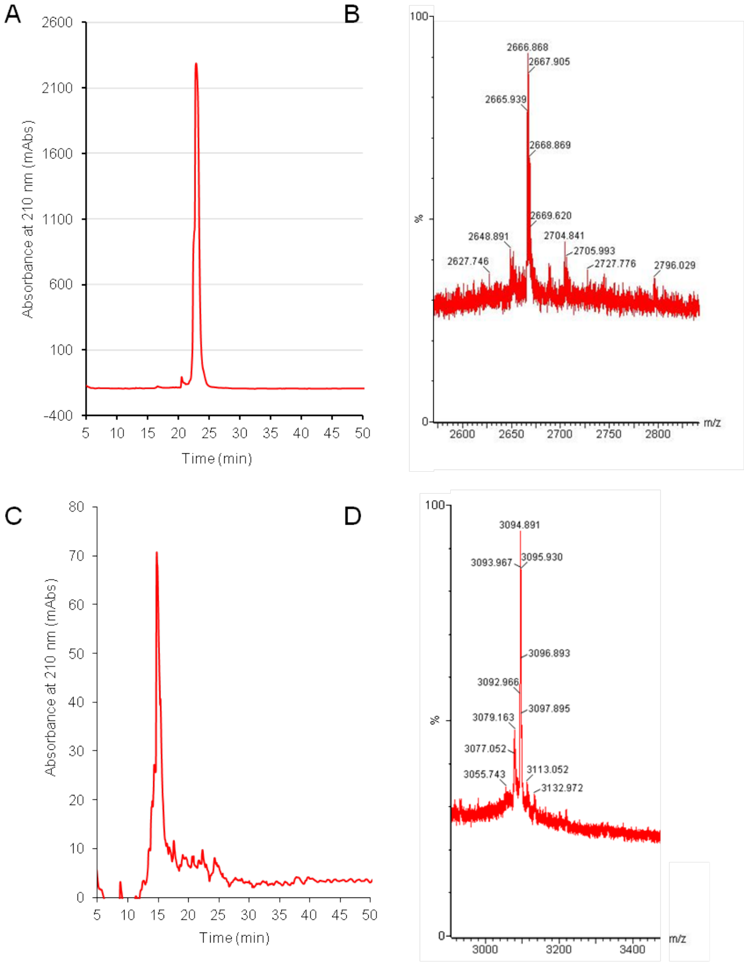

Figure S1: A) *Analytical HPLC of LOV-BID peptide* B) *MALDI-TOF spectrum of LOV-BID peptide showing a peak at m/z 2666 ([M+H]$^+$)* C) *Analytical HPLC of FAM-LOV-BID peptide* D) *MALDI-TOF spectrum of FAM-LOV-BID peptide at 3094.8 m/z ([M+H]$^+$).*

Table S3: *Calculated monoisotopic masses and observed masses of BID-BH3 and LOV-BID peptides.*

Peptide	Sequence	Calculated monoisotopic mass	Observed mass ([M+H]$^+$)
BID BH3	CGDIIRNIARHLAQVGDSIDRSI-NH_2	2520.3	2521.9
TMR-BID BH3		3003.4	3004.2
LOV-BID	DCAEDIGVNIARHLAQVGDSIDRSI-NH_2	2665.0	2666.0
FAM-LOV-BID		3093.4	3094.8

Fluorescence anisotropy measurements were performed at 15 °C on a Perkin Elmer LS55 luminescence spectrometer arranged in L format (FAM: 492 nm excitation, 520 nm emission TAMRA: 545 nm excitation, 573 nm emission). A quartz fluorescence cuvette (4 mL) contained labelled protein or peptide (2.5-10 nM) in sodium phosphate buffer (3 mL, 50 mM, pH 7.5) containing sodium chloride (10 mM). Bak peptide, Bcl-x_L solutions at concentrations of 0-5000 nM were successively added to cuvette. For peptide-peptide or peptide-protein assays each anisotropy value was from twenty single measurements

were taken using an integration time of 1 sec. For protein-protein binding assays each anisotropy value was from ten single measurements taken using an integration time of 5 sec. The G factor (ratio of sensitivities of the monochromator for horizontally and vertically polarised light) can be calculated using the equation:

$$G = \frac{I_{HV}}{I_{HH}}$$

Where, I_{HH} and I_{HV} are the intensities of the fluorescent emissions in parallel and perpendicular planes, respectively to the excitation plane. The G factor value was always close to 1.16 for FITC-labelled peptides and 0.85 for TMR-labelled proteins. Values for fluorescence anisotropy (*A*) were then determined from the equation:[3]

$$A = I_{HH} - \frac{GI_{HV}}{I_{HH}} + 2GI_{HV}$$

The data were fit to the Langmuir isotherm:

$$\emptyset_{Fit} = \frac{1}{1 + \left(\frac{{K_D}^n}{[P]^n}\right)}$$

Where, $\emptyset_{Fit}$ denotes the fraction of bound labelled-ligand, K_D is the apparent dissociation constant, [P] is the concentration of protein and *n* is the number of binding sites. All binding curves were acquired independently at least three times and the resulting K_D values averaged. Errors are the standard errors of the mean for each concentration point. Fluorescence anisotropy data were normalised and expressed as:

$$F = \frac{(A - A_D)}{(A_{max} - A_D)}$$

Where, *A* denotes the fluorescence anisotropy in the presence of the indicated concentration of protein, A_D denotes the fluorescence anisotropy in the absence of protein, and A_{max} denotes the fluorescence anisotropy at saturation.

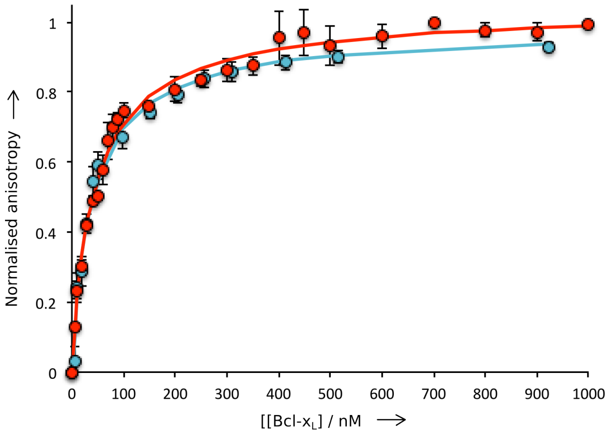

Figure S2: *Normalised fluorescence anisotropy binding curves for Bcl-x$_L$ titrated into TMR-BID BH3 (5 nM, red) B) FAM-LOV-BID peptide (10 nM, blue) in sodium phosphate buffer (50 mM, pH 7.5) containing sodium chloride (10 mM).*

Table S4: *Binding affinities of BID-BH3 and LOV-BID Jα peptides to Bcl-x_L determined by fluorescence anisotropy.*

Peptide	*Sequence*	*k_D (nM)*
BID-BH3	CGDIIRNIARHLAQVGDSIDRSI-NH_2	27 ± 5
Jα–BID	DCAEDIGVNIARHLAQVGDSIDRSI-NH_2	46 ± 3

3 pNCO-Hisactophilin (C49S)-*AS*LOV-BID1

The plasmid harbouring the gene encoding Hisact-*AS*LOV (GenBank EF493211) was modified according to the Quikchange protocol.

Primers to introduce V416I:

Fwd:

GAACGTATTGAGAAGAACTTTATTATTACTGACCCACGTTTGCC

Rev:

GGCAAACGTGGGTCAGTAATAATAAAGTTCTTCTCAATACGTTC

The resulting plasmid harbouring the gene encoding Hisact-*AS*LOV-V183I contains *Kpn*I and *Hin*dIII restriction sites (Figure 4.8), which were used to excise the wild-type Jα-helix. The doubly cut plasmid was purified by agarose gel electrophoresis (Figure 4.9). Annealed oligonucleotides with complimentary sticky ends encoding the hybrid Jα (LOV-BID) were ligated with the purified linearised pNCO plasmid DNA. *E. Coli* XL-1 Blue cells were transformed with the resulting DNA and individual colonies were grown in overnight tubes and their DNA sequenced until the intended plasmid was isolated.

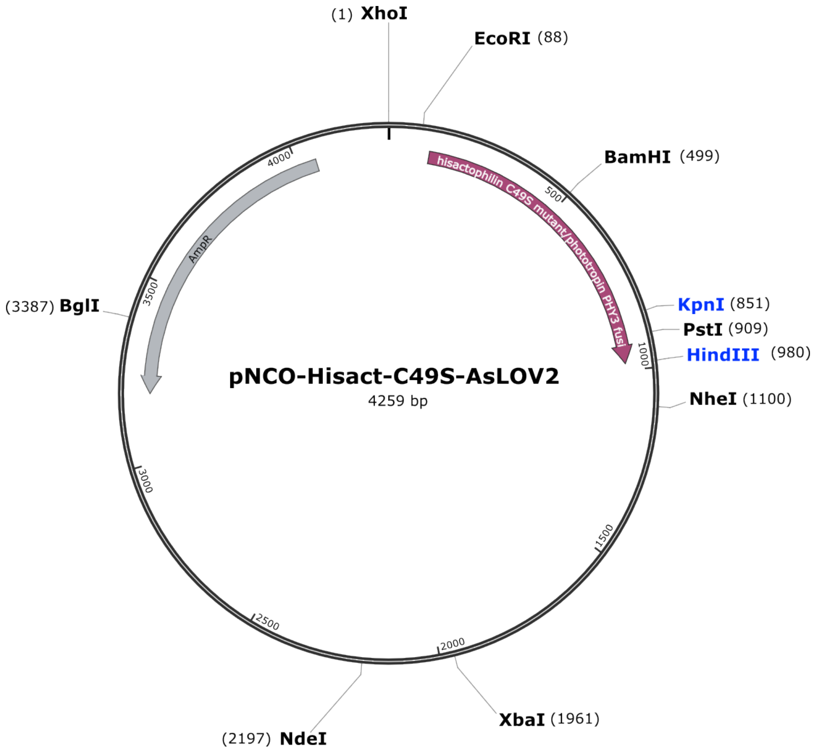

Figure S3: *pNCO-Hisact-ASLOV2-V183I vector map. KpnI and HindIII sites are marked in blue.*

Primers to create Hisact(C49S)-*AS*LOV-BID1:

Fwd 1:

CGAACATGTCCGTGATTGCGCCGAGGATTGGTGTCAACATCGCGCGTCATCTGGCACAGGTGGGTGAT

Fwd2:

AGCATTGATAGCCGTATTCCAGATGCTAATCTGCGTCCAGAGGATTTGTGGGCTAACTA

Rev1:

AGCTTAGTTAGCCCACAAATCCTCTGGACGCAGATTAGCATCTGGAATACGGCTATCAATGCTATCACCCACCT
GTGC

Rev2:

CAGATGACGCGCGATGTTGACACCAATATCCTCGGCGCAATCACGGACATGTTCGGTAC

E. coli BL21 (DE3) *Star* cells were transformed with this DNA and grown to OD_{600} 0.8. Protein production was induced by the addition of IPTG and expression was allowed to continue for overnight at 20 °C. Over-expression of a protein migrating at the expected speed was verified by SDS-PAGE. The protein was purified by Ni-NTA affinity followed by purification by size-exclusion chromatography.

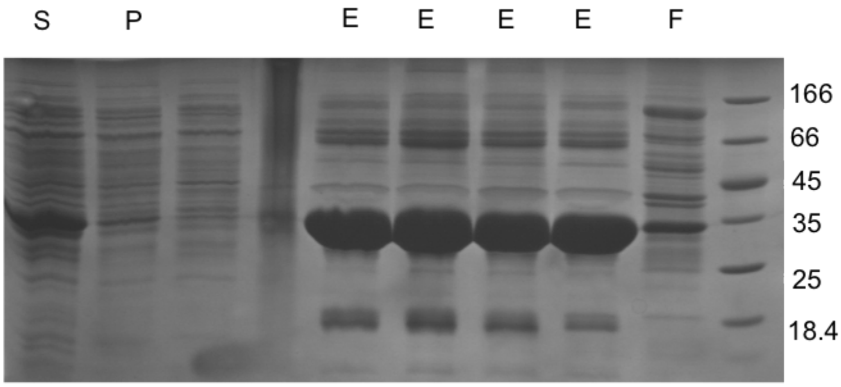

Figure S4: *Purification of pNCO-Hisact-ASLOV-V183I-BID showing cell lysate supernatant (S), pellet (P), Ni-NTA column flow through (F) and fractions eluted with imidazole (E).*

Table S5: *Half-lives of Hisactophilin-LOV fusion proteins by UV/Vis recovery at 450 nm and circular dichroism (CD) spectrometry at 222 nm.*

Protein	*Half-life (min)*	
	$t_{1/2}^{UV}$	$t_{1/2}^{CD}$
Hisactophilin-*AS*LOV2	0.99 ± 0.05	N/D [b]
Hisactophilin-*AS*LOV-V183I	7.69 ± 0.10	11.1 ± 2.70
Hisactophilin-*AS*LOV-BID1[a]	8.85 ± 0.10	11.3 ± 3.23

[a] All LOV-BID proteins described include the V416I mutation.
[b] This result is difficult to measure as the sample transfer time from LED to CD spectrometer and spectrometer initialisation is significant on this timescale.

4 LOVBID1-4

In order to more conveniently isolate the *ASLOV*-BID1 from the hisactophillin domain, *Nde*I and *Bam*HI restriction sites were incorporated at the start and end of the LOV-BID sequence *via* site-directed mutagenesis and the confirmed by sequencing. The gene encoding LOV-BID (474 bp) was then cut at these sites and the resulting sticky-ends were ligated to multiple cloning site of pET28a doubly cut using the same restriction enzymes. The resulting DNA was used to transform super-competent *E. Coli* XL1-Blue cells that were incubated on an agar plate containing kanamycin overnight. Overnight cultures were inoculated with single colonies from the plate, harvested and DNA extracted until a digestion test was performed in order to test whether the insert had been ligated into the vector (Figure 4.15). The pET28a vector does not contain a *Kpn*I restriction site whereas the LOVBID sequence does, therefore digested by *Kpn*I (5.4 kbp vector plus 474 bp insert) were sequenced to confirm the presence of the desired gene.

The pET28a plasmid harbouring the gene encoding *AS*LOV-BID1 retains the *Kpn*I and *Hin*dIII restriction sites used to insert the original BID sequence and these were used once again to excise the *AS*LOV-BID1 Jα-helix in order to insert annealed oligonucleotides encoding further the hybrid Jα sequences (LOVBID2-4)

<u>LOV-BID2</u>
Fwd 1:
CGAACATGTCCGTGATGCGGCCGAGCGTGAGGGTGTC
Fwd2:
ATGCTGATTAAGGATATTGCACGTAATATTGATCGT GCG
Fwd3:
CTGGCGGAAGTGGGTGATAGCATTGATCGTAGCATTTA
Rev1:
AGCTTAAATGCTACGATCAATGCTATCACCCAC
Rev2:
TTCCGCCAGCGCACGATCAATATTACGTGCAATATCCTT
Rev3:
AATCAGCATGACACCCTCACGCTCGGCCGCATCACGGACATGTTCGGTAC

<u>LOV-BID3</u>
Fwd1:
CGAACATGTCCGTGATGCGGCCGAGCGTGAGGGTGTC
Fwd2:
ATGCTGATTAAGAAAACTGCAGATATTATTGATAACGCGGCACGT
Fwd3:
GAACTTGCACAGGTGGGTGATAGCATTGATCGTAGCATTTA
Rev1:
AGCTTAAATGCTACGATCAATGCTATCACCCAC
Rev2:
CTGTGCAAGTTCACGTGCCGCGTTATCAATAATATCTGCAGTTTTCTT
Rev3:
AATCAGCATGACACCCTCACGCTCGGCCGCATCACGGACATGTTCGGTAC

<u>LOV-BID4</u>
Fwd1:
CGAACATGTCCGTGATGCGGCCGAGCGTGAGGGTGTC
Fwd2:
ATGCTGATTAAGAAAACTGCAGAAAATGATATTGCGCGTAATATC
Fwd3:
GCGCGTCATCTGGCACAGGTGGGTGATAGCATTGATCGTAGCATTTA
Rev1:
AGCTTAAATGCTACGATCAATGCTATCACCCACCTGTGC
Rev2:
CAGATGACGCGCGATATTACGCGCAATATCATTTTCTGCAGTTTTCTT
Rev3:
AATCAGCATGACACCCTCACGCTCGGCCGCATCACGGACATGTTCGGTAC

The resulting plasmids were used to transform super competent XL1-Blue cells that were plated on selective agar media. Individual colonies were picked and their DNA sequenced. Sequenced DNA was used to transform BL21(DE3)-Star cells for protein expression. Cells were grown in TB media at 37 ^{o}C until OD_{600} = 0.6 then induced with IPTG (0.5 mM) and shaken at 20 ^{o}C overnight. The cells were harvested and lysed using an ultrasonicator (3 seconds on and 5 seconds off for 6 minutes) then resulting lysate was centrifuged and the cell lysate and pellet were analysed by SDS-PAGE. All the proteins were present in the supernatant of the cell lysate, additionally LOVBID1 and LOVBID2 contained protein in the pellet of cell debris. Proteins were purified by Ni-NTA affinity chromatography and size-exclusion chromatography and the masses were determined by ESI-TOF MS on a Waters Q-TOF. SDS-PAGE analysis of eluted fractions from preparative Superdex 75 column, show single bands for *AS*LOV-BID1-4 at the approximately the correct migration speed. All four observed protein masses deviate from the theoretical monoisotopic masses (including FMN at 456.34 Da) by less than 0.5 %.

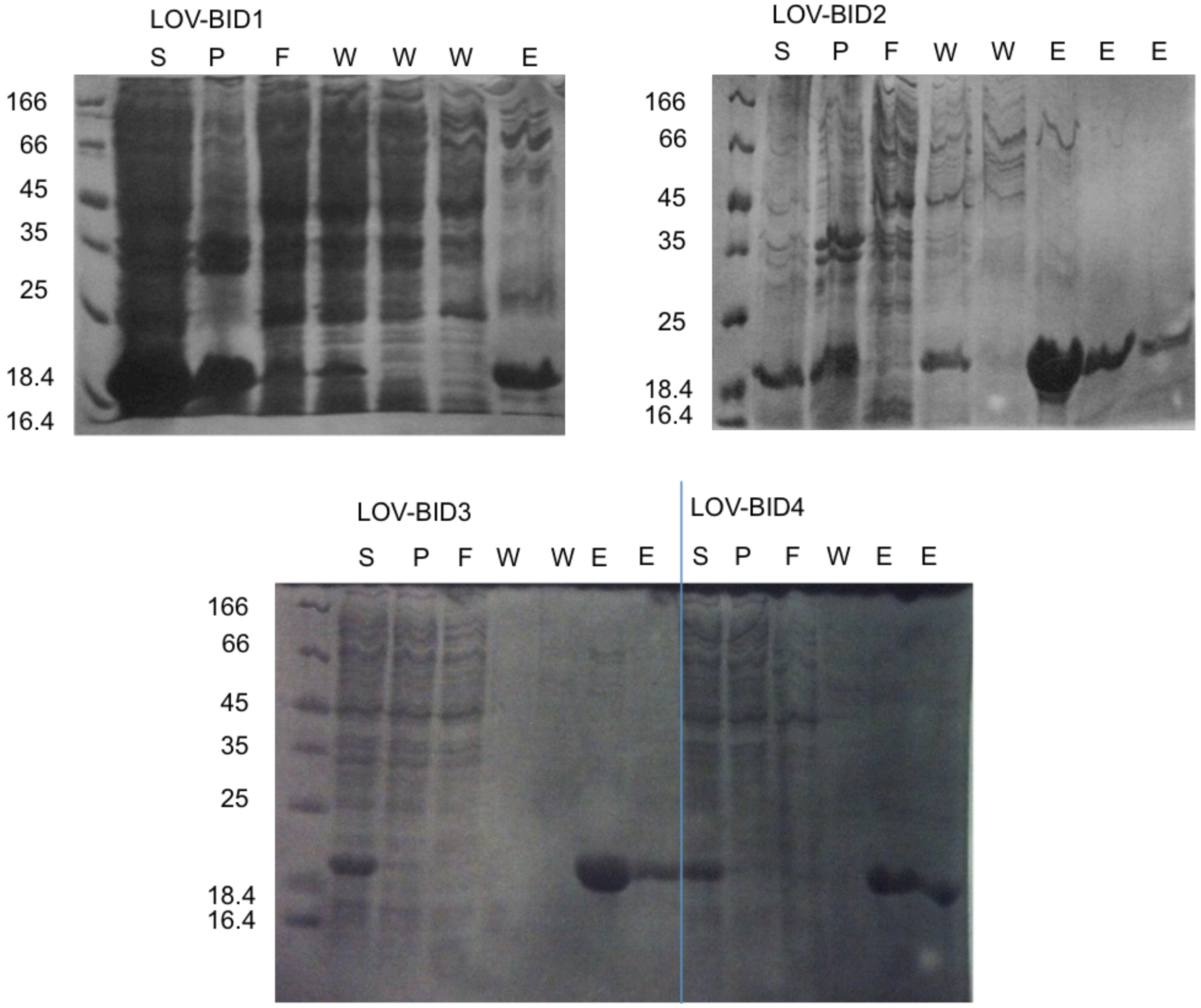

Figure S5: SDS-PAGE gels of cell lysate and Ni-NTA purification for LOV-BID1-4.

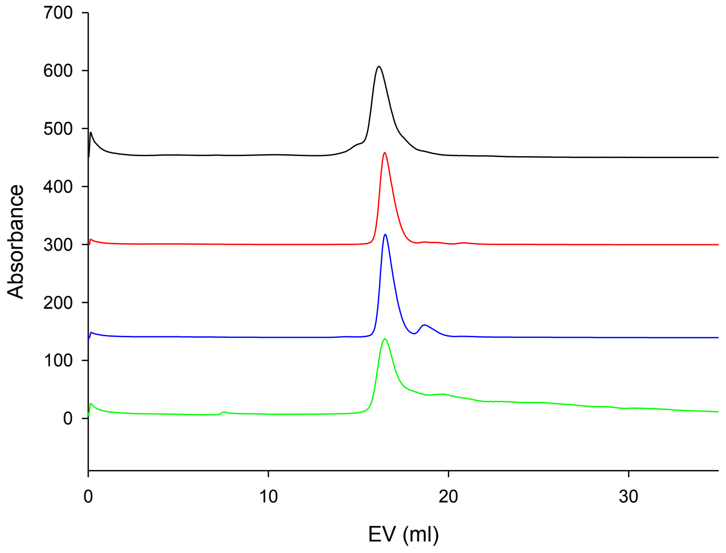

Figure S6: *Size-exclusion chromatographs of LOV-BID1 (black), LOV-BID2 (red), LOV-BID3 (blue) and LOV-BID4 (green) run on an analytical Superdex 200 column.*

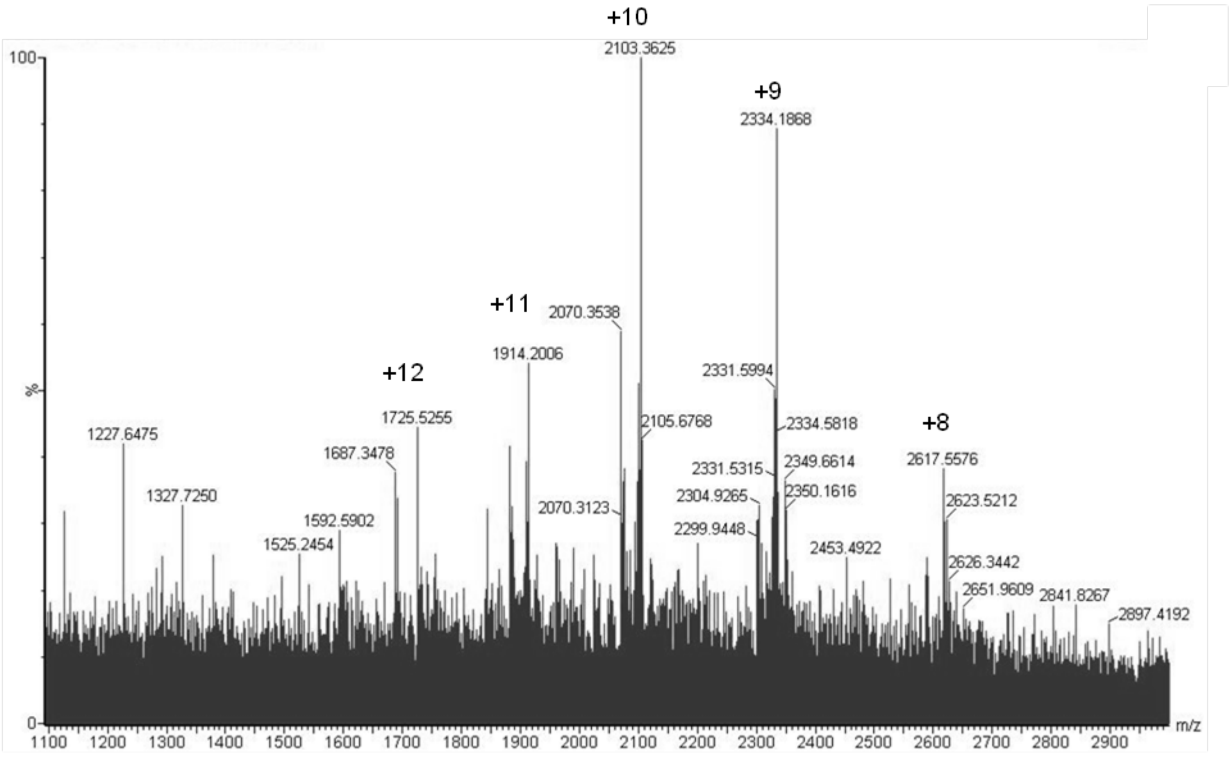

m/z	net charge	MW (Da)		MW (Da)
1725.5255	+12	20694.306	*Calculated*	20938.856
1914.2006	+11	21045.2066	*Theoretical*	20812.569
2103.3625	+10	21023.625	*Difference*	126.287
2334.1868	+9	20998.6812		
2617.5576	+8	20932.4608	*% Difference*	0.60

Figure S7: ESI-TOF spectrum and analysis for LOV-BID1.

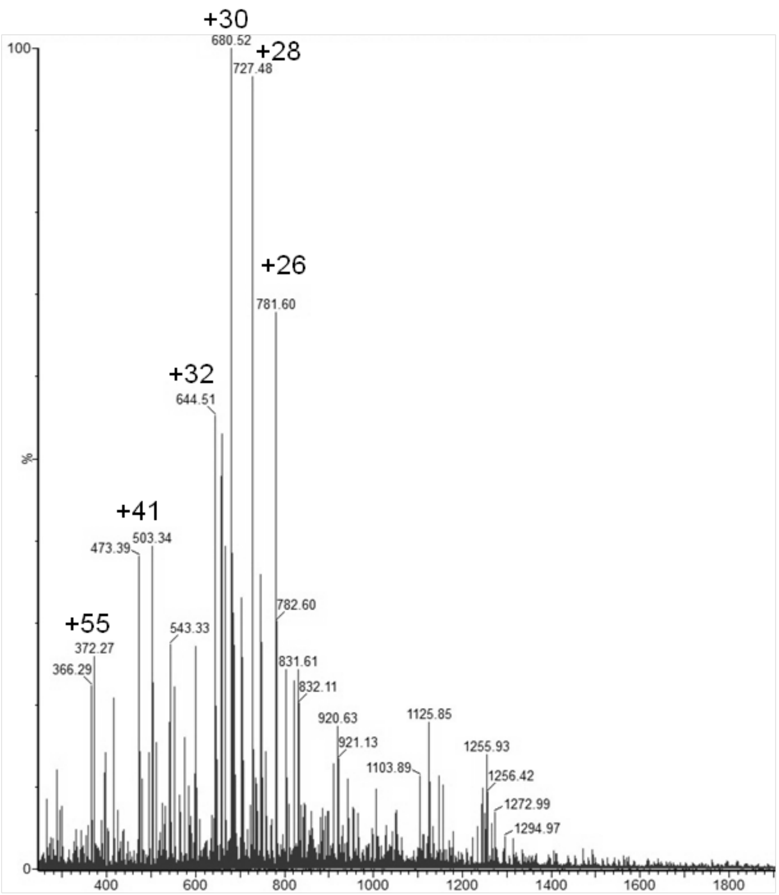

m/z	net charge	MW (Da)		MW (Da)
372.27	+55	20419.9	*Calculated*	20438.458
503.34	+41	20595.9	*Theoretical*	20523.535
644.51	+32	20592.3	*Difference*	85.077
680.52	+30	20385.6		
727.48	+28	20341.4	*% Difference*	0.41
781.6	+26	20295.6		

Figure S8: ESI-TOF spectrum and analysis for LOV-BID2.

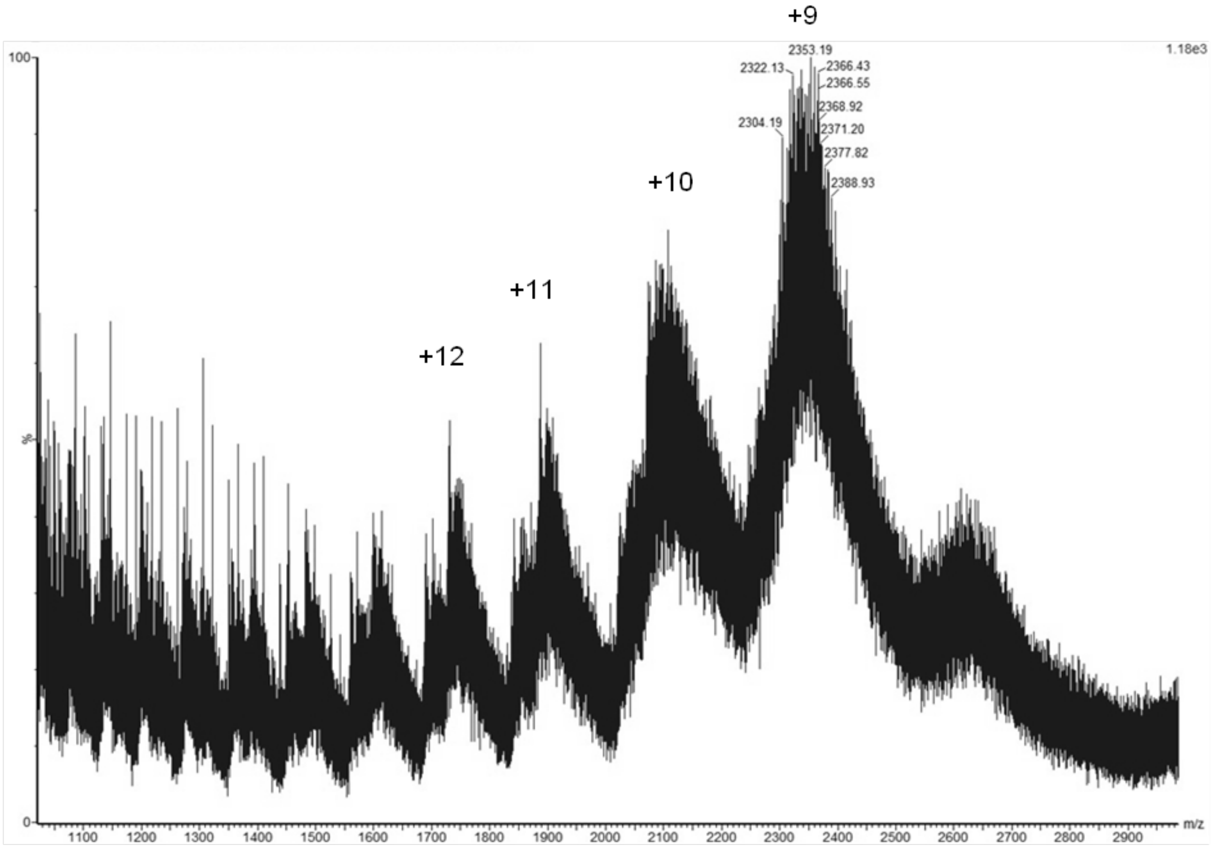

m/z	net charge	MW (Da)		MW (Da)
1731.94	+12	20771.3	*Calculated*	20940.9
1887.47	+11	20751.2	*Theoretical*	20928.8
2108.14	+10	21071.4	*Difference*	12.2
2353.19	+9	21169.7	*% Difference*	0.058

Figure S9: ESI-TOF spectrum and analysis for LOV-BID3.

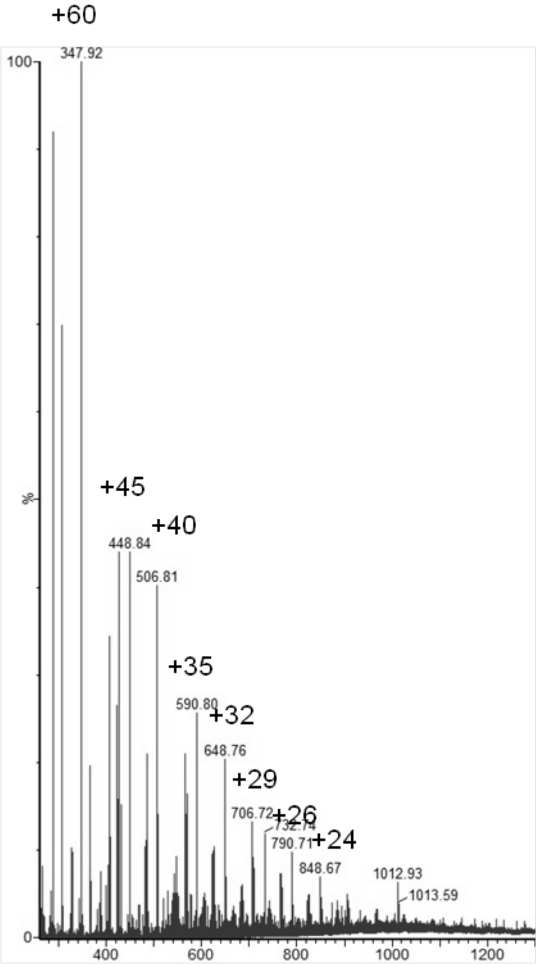

m/z	net charge	MW (Da)		MW (Da)
347.92	+60	20815.2	*Calculated*	20489.27
448.84	+45	20152.8	*Theoretical*	20523.55
506.81	+40	20232.4	*Difference*	34.2825
590.8	+35	20643		
648.76	+32	20728.3	*% Difference*	0.167
706.72	+29	20465.9		
790.71	+26	20532.5		
848.67	+24	20344.1		

Figure S10: ESI-TOF spectrum and analysis for LOV-BID4.

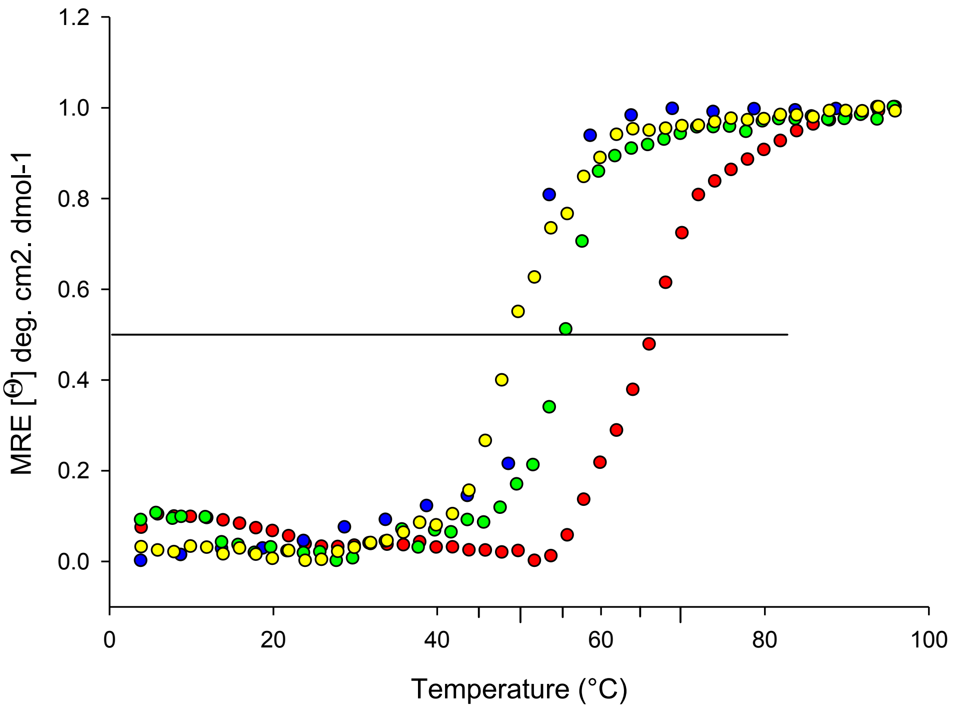

Figure S11: *Normalised plots of mean residue ellipticity 222 nm of LOV-BID1 (red) LOV-BID2 (green) LOV-BID3 (blue) and LOV-BID4 (yellow) over a temperatures range from 4 to 95 °C in 50 mM sodium phosphate buffer (pH 7.5) containing sodium chloride (10 mM).*

Table S5: *Half-lives of Hisactophilin-LOV fusion proteins by UV/Vis recovery at 450 nm and circular dichroism (CD) spectrometry at 222 nm.*

Protein [a]	Half-life (min)	
	$t_{1/2}^{UV}$	$t_{1/2}^{CD}$
His$_6$*AS*LOV2-V416I	11.4 ± 0.12	13.0 ± 2.80
LOV-BID1	10.4 ± 0.05	12.3 ± 2.40
LOV-BID2	8.60 ± 0.05	10.3 ± 3.05
LOV-BID3	7.50 ± 0.17	7.50 ± 1.00
LOV-BID4	7.80 ± 0.75	7.80 ± 0.80

[a] All LOV-BID proteins described include the V416I mutation to stabilise the cysteinyl-FMN adduct.

5 Bcl-x$_L$ S2C

The following primers were used to perform site directed mutagenesis on a *p*ET21a plasmid encoding Bcl-x$_L$ (1-209 Δ45-84, Δ210-233) to effect a S2C mutation using *Pfu* polymerase (ThermoFisher Scientific) according to the manufacturers instructions.

Fwd:
GGAGATATACATATGTGCCAGTCTAACCGTG
Rev:
CACGGTTAGACTGGCACATATGTATATCTCC

Once the reaction was complete *Dpn*I (1 μl, 20 units) was added to each of the samples which were then incubated for 1.5 hours at 37°C to destroy template plasmid. The products were stored at -20 °C, then used to transform supercompetent *E. coli* XL1-Blue. Transformation mixtures were plated on LB agar containing kanamycin. Overnight incubations (10 mL LB media) were grown from single colonies and their DNA isolated using a QIAQuick miniprep kit. A sample that produced a satisfactory DNA sequencing result was used to transform *E. coli* BL21 DE3 cells for expression. Chemically competent cells were transformed with plasmid DNA, plated onto selective media and grown overnight at 37 ^{o}C. Individual colonies were then picked and grown overnight in LB medium (100 mL) containing the kanamycin (5 mg). Samples from the overnight culture were transferred to fresh LB medium (5 mL inoculum per 500 mL) containing kanamycin (50 mg/mL) in 2 L conical flasks, and incubated until they reached an OD_{600} of 0.6-0.8. The cells were then induced by the addition of IPTG to a final concentration of 1 mM, and left to grow for 4 to 6 hours. The cells were then centrifuged at 6,000 *g* (Sorvall RC6 Plus) for 30 minutes at 4 °C and pellets were stored at -20 °C.

For fluorescent labelling, protein samples were dialysed into Tris (50 mM, pH 8.3) buffer containing sodium chloride (100 mM) with triscarboxyethylphosphine (TCEP, 2 mM, added from 100 mM stock) reducing agent and incubated with maleimidotetramethylrhodamine at 15 °C overnight for proteins mixing at 300 rpm (Eppendorf Thermo Mixer Comfort). The protein samples were then dialysed into 50 mM disodium phosphate buffer pH 7.5 to remove excess dye, followed by size exclusion chromatography eluting in 50 mM sodium phosphate buffer (pH7.5) containing 100 mM sodium chloride and 5 mM β-mercaptoethanol.. An approximate extinction co-efficient of 91,000 $M^{-1}cm^{-1}$ at 551 nm was used to estimate the concentration of labelled protein. Bcl-x_L-TAMRA conjugation was quantified by comparing the labelled Bcl-x_L-S2C(TMR) concentration with the total Bcl-x_L-S2C concentration before reaction using an extinction co-efficient of 41,940 $M^{-1}cm^{-1}$ at 280 nm and ≥90 % labelled protein samples were prepared for fluorescent anisotropy measurements.

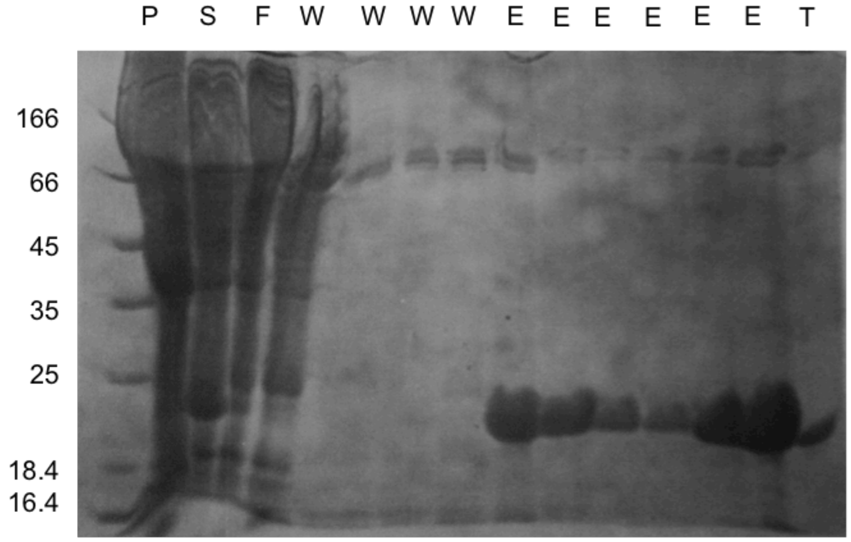

Figure S12: 12 % *SDS-PAGE gel of Bcl-x_L-S2C purification. Showing cell lysis pellet (P), supernatant (S), Ni-NTA flow through (F), washes (W), imidazole elutions (E) and TAMRA labelled protein (T).*

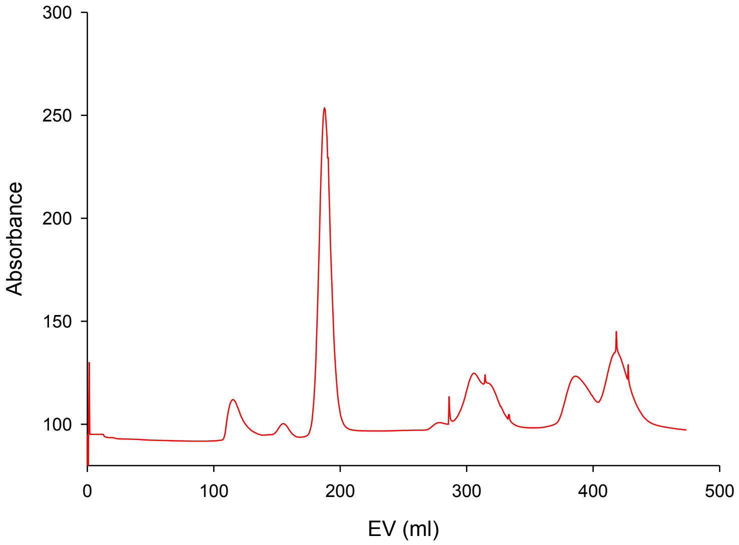

Figure S13: *Size-exclusion chromatogram of TAMRA labelled Bcl-x$_L$-S2C(TMR) (190 mL) and excess TAMRA maleimide (290 ml).*

Fluorescence anisotropy measurements between the *AS*LOV-BID domains and TMR-labelled Bcl-x$_L$ were carried out at a reduced temperature (15 ^{o}C) to minimise reversion to the dark-state during the recording of individual data points and titrations were repeated at least in triplicate.

6 References

[1] D. Strickland, Y. Lin, E. Wagner, C. M. Hope, J. Zayner, C. Antoniou, T. R. Sosnick, E. L. Weiss, M. Glotzer, *Nat. Meth.* **2012**, *9*, 379-384.

[2] O. I. Lungu, R. A. Hallett, E. J. Choi, M. J. Aiken, K. M. Hahn, B. Kuhlman, *Chem. Biol.* **2012**, *19*, 926-926.

[3] G. Guntas, R. A. Hallett, S. P. Zimmerman, T. Williams, H. Yumerefendi, J. E. Bear, B. Kuhlman, *Proc. Natl. Acad. Sci. USA* **2015**, *112*, 112-117.

[4] J. J. Yi, H. Wang, M. Vilela, G. Danuser, K. M. Hahn, *ACS Synth. Biol.* **2014**, *3*, 788-795.

[5] K. M. Bonger, R. Rakhit, A. Y. Payumo, J. K. Chen, T. J. Wandless, *ACS Chem. Biol.* **2014**, *9*, 111-115.
